# COVID-19: underpowered randomised trials, or no randomised trials?

**DOI:** 10.1186/s13063-021-05209-5

**Published:** 2021-03-29

**Authors:** Atle Fretheim

**Affiliations:** 1 Centre of Informed Health Choices, Norwegian Institute of Public Health, Oslo, Norway; 2 Faculty of Health Sciences, Oslo Metropolitan University, Oslo, Norway

## Abstract

A recently published trial of face mask use to protect against COVID-19 demonstrated a key barrier to carrying out randomised trials in public health: the need for unattainably large sample sizes. For many public health interventions, the choice is not between sufficiently powered trials and underpowered trials, but between underpowered trials and no trials at all. Underpowered trials should be viewed as contributions to the larger body of evidence, alongside other studies of various sizes and designs, collectively assessed and synthesized in systematic reviews. Overemphasis on sample size calculation is probably more of a hindrance than a help to scientific progress.

## Background

The recently published Danish trial of face masks for COVID-19 prevention [1] has spurred controversy [2–4]. A major criticism is that the trial was powered for an overly large effect size, i.e. the researchers based their sample size calculation on the assumption that a simple recommendation to use masks for protection outdoors, would halve the risk of infection.

Consequently, the study was underpowered: the 95% confidence interval around the modest 18% relative risk reduction in the face mask group included both a substantial effect (46% relative risk reduction), as well as an increased risk of infection (23% relative risk increase). Consequently, the trial results are of limited direct value for decision makers.

So, was the trial a waste of time?

## Few trials in public health

The COVID-19 pandemic serves as an illustration of a well-known problem: with the notable exception of vaccines, randomised trials of public health interventions are rare. The Danish face mask trial is one of very few conducted trials of interventions to curb the spread of SARS-CoV-2—in stark contrast to the abundance of trials of pharmaceuticals and other clinical interventions for COVID-19 [5].

The need for large sample sizes is a key barrier to carrying out randomised trials in public health, mainly due to low event rates. Even during the most intense phases of the COVID-19 pandemic, weekly incidence rates have rarely exceeded 1% in the general population—e.g. for England, the highest ever recorded is 0.7% [6]. Demonstrating a risk reduction from, say, 1.2% to 0.8% requires 20,000 participants. When we planned a randomized trial to evaluate the effect of school closures to limit the spread of the virus, the results of our sample size calculations meant that we needed to enroll nearly all schools in Norway [7].

In practice, we are often not able to choose between having sufficiently powered trials and underpowered trials. The realistic choice is between underpowered trials and no trials at all.

Surely, some trial evidence must be better than no trial evidence?

## The larger body of evidence, not individual trials

The main risk with underpowered trials is that of type II errors, i.e. the study findings may be too imprecise to demonstrate an important, but real effect. The Danish face mask trial may be an example of that. Further, when trials do not provide clear evidence of an effect, the results may be erroneously interpreted as evidence of no effect. A recent example is from a headline reporting on the Danish study: “Face masks do NOT protect the wearer from coronavirus” [8].

Another challenge is it may be considered unethical to recruit participants to a study that is unlikely to yield conclusive results [9].

However, the idea that a single trial provides a definitive answer should have been abandoned long ago. Underpowered trials should be viewed as contributions to the larger body of evidence, alongside other studies of various sizes and designs, collectively assessed and synthesized in systematic reviews [9]. Pooling of findings from well-conducted but small trials in a meta-analysis can yield statistically robust results.

Face mask use in the community has been evaluated previously, e.g. in two trials with American college students during influenza seasons [10, 11]. The findings from those studies—both with inconclusive results—are included in the body of evidence that has informed recommendations about face mask use during the COVID-19 pandemic [12, 13]. The Danish face mask supplements the earlier trial findings and demonstrates that it is feasible to conduct such trials during a pandemic.

Another reason to tone down the emphasis on sample size calculations, in general, is that researchers would be less incentivised to game the system by adjusting assumptions about expected effect sizes and event rates in order to arrive at feasible sample sizes [9]. It might also discourage the use of composite endpoints or other inventions that mainly serve the purpose of artificially inflating the statistical power of trials [9].

## Conclusion

Sufficiently powered trials are certainly preferable to underpowered ones, but overemphasis on sample size calculation is probably more of a hindrance than a help to scientific progress.

Underpowered trials should be viewed as contributions to the larger body of evidence, alongside other studies of various sizes and designs, collectively assessed and synthesized in systematic reviews.

## Data Availability

Not applicable
